# Author reply to “Being a family medicine resident in the United States”

**DOI:** 10.1002/jgf2.403

**Published:** 2020-11-19

**Authors:** Brian S. Heist, Haruka Matsubara Torok

**Affiliations:** ^1^ Division of General Internal Medicine School of Medicine University of Pittsburgh Pittsburgh PA USA; ^2^ University of Minnesota Medical School Minneapolis MN USA

## Abstract

This is a reply to a Letter to the Editor regarding our recent publications. We feel that the letter's content largely aligns with the content of our work.

To the Editor,

We are grateful for Dr Kuroda's thoughtfully written letter^1^ that will hopefully enhance other readers' understanding of the US clinical training experience. A main objective of these publications^2^, ^3^ is to assist Japanese physicians who aspire to US clinical training in their decision‐making and how to be successful.

It is our impression that Dr Kuroda's pathway into and through US clinical training resonate with findings from our work, particularly the experiences with the English language and ultimate sentiment that the struggles and effort were worth it.

Dr Kuroda describes personal experiences during US family medicine residency, which are thus more pertinent to the article, “Japanese International Medical Graduates and the United States clinical training experience: Challenges abroad and methods to overcome them.”^3^ We believe that the first two benefits of US family medicine training asserted by the letter articulate examples of the article's themes of exposure to socioeconomic and cultural diversity, and learning evidence‐based medicine. We appreciate all of Dr Kuroda's reflections. The third additional benefit of US training stated by the letter addresses opportunity within US family medicine residency to study comprehensive and longitudinal patient care that contrasts the common Japanese model. Our work was not focused on family medicine, and in turn, we strove to identify themes of training in the United States that span clinical disciplines. Notably, our study participants provided rich descriptions of numerous elements of the Japanese IMG experience, some of which proved relevant to the international medical educator community. Our articles on Japanese IMGs and the reformation of generalist fields in Japan^4^ cited by Dr Kuroda and on the atypical experience of Japanese compared to other IMGs in the United States^5^ recognize the value of training in family medicine in the United States where the field is more developed and integrated into the organization of healthcare and, separately, the impact of the English language barrier on the expansion of Western practices, including EBM, within Japan.

We again thank Dr Kuroda for the personal reflections that help to further depict the Japanese IMG experience.

## CONFLICTS OF INTEREST

The authors declare they have no competing interests.
